# Local-duality QCD sum rules for strong isospin breaking in the decay constants of heavy–light mesons

**DOI:** 10.1140/epjc/s10052-018-5637-z

**Published:** 2018-02-24

**Authors:** Wolfgang Lucha, Dmitri Melikhov, Silvano Simula

**Affiliations:** 1 Institute for High Energy Physics, Austrian Academy of Sciences, Nikolsdorfergasse 18, 1050 Vienna, Austria; 2 D. V. Skobeltsyn Institute of Nuclear Physics, M. V. Lomonosov Moscow State University, Moscow, 119991 Russia; 3 Faculty of Physics, University of Vienna, Boltzmanngasse 5, 1090 Vienna, Austria; 4 Istituto Nazionale di Fisica Nucleare, Sezione di Roma Tre, Via della Vasca Navale 84, 00146 Rome, Italy

## Abstract

We discuss the leptonic decay constants of heavy–light mesons by means of Borel QCD sum rules in the local-duality (LD) limit of infinitely large Borel mass parameter. In this limit, for an appropriate choice of the invariant structures in the QCD correlation functions, all vacuum-condensate contributions vanish and all nonperturbative effects are contained in only one quantity, the effective threshold. We study properties of the LD effective thresholds in the limits of large heavy-quark mass $m_Q$ and small light-quark mass $m_q$. In the heavy-quark limit, we clarify the role played by the radiative corrections in the effective threshold for reproducing the pQCD expansion of the decay constants of pseudoscalar and vector mesons. We show that the dependence of the meson decay constants on $m_q$ arises predominantly (at the level of 70–80%) from the calculable $m_q$-dependence of the perturbative spectral densities. Making use of the lattice QCD results for the decay constants of nonstrange and strange pseudoscalar and vector heavy mesons, we obtain solid predictions for the decay constants of heavy–light mesons as functions of $m_q$ in the range from a few to 100 MeV and evaluate the corresponding strong isospin-breaking effects: $f_{D^+} - f_{D^0}=(0.96 \pm 0.09) \ \mathrm{MeV}$, $f_{D^{*+}} - f_{D^{*0}}= (1.18 \pm 0.35) \ \mathrm{MeV}$, $f_{B^0} - f_{B^+}=(1.01 \pm 0.10) \ \mathrm{MeV}$, $f_{B^{*0}} - f_{B^{*+}}=(0.89 \pm 0.30) \ \mathrm{MeV}$.

## Introduction

The method of QCD sum rules [1], based on the exploitation of Wilson’s operator product expansion (OPE) in the study of properties of individual hadrons, has been extensively applied to the decay constants of heavy mesons [2–4]. An important finding of these analyses was the observation of the strong sensitivity of the decay constants to the precise values of the input OPE parameters and to the algorithm used for fixing the effective threshold [5–9]. For any given approximation of the hadronic spectral density based on quark–hadron duality, the effective threshold determines to a large extent the numerical prediction for the decay constants inferred from QCD sum rules: even if the parameters of the truncated OPE are known with high precision, the decay constants may be predicted with only a limited accuracy, which represents their systematic uncertainty. In a series of papers [10–14], we proposed a new algorithm for fixing the effective threshold within the Borel QCD sum rules which allowed us to obtain realistic estimates of the systematic uncertainties. Our procedure opened the possibility to get predictions for the decay constants with a controlled accuracy [15–18] and thus allowed us to address subtle effects that call for a profound accurate treatment, such as the ratios of the decay constants of heavy vector and pseudoscalar mesons [19, 20] or the strong isospin-breaking (IB) effects in the decay constants of heavy–light mesons [21], generated by the mass difference ($m_d - m_u$) between up and down quarks.

Here, we discuss the application of another variant of QCD sum rules to the evaluation of the strong IB effects in the decay constants of heavy–light pseudoscalar and vector mesons. Our analysis takes advantage of the fact that the OPE provides the analytic dependence of the correlation functions on the quark masses; this allows us to study, e.g., the impact of the light-quark mass on heavy-meson decay constants, thus providing access to the strong IB effects. The approach we describe in this work seems quite promising for studying the dependence of a generic hadron observable on quark masses.

### QCD sum rule in the local-duality limit

A typical Borel QCD sum rule for the decay constant $f_H$ of a heavy (pseudoscalar or vector) $\bar{Q}q$ meson *H* of mass $M_H$, consisting of a heavy quark *Q* with mass $m_Q$ and a light quark *q* with mass $m_q$, has the form

1 $$\begin{aligned}&f_H^2 (M_H^2)^N \mathrm{e}^{-M_H^2 \tau } \nonumber \\&\quad =\int \limits _{(m_Q+m_q)^2}^{s^{(N)}_\mathrm{eff}(\tau , m_Q, m_q, \alpha _s)}\mathrm{d}s \mathrm{e}^{-s \tau } s^N \rho _\mathrm{pert}(s, m_Q, m_q, \alpha _s) \end{aligned}$$

Here, $\tau$ is the Borel parameter, *N* is an integer that depends on the Lorentz structure in the correlation function chosen for the sum rule and on the number of subtractions in the corresponding dispersion representation, and $s_\mathrm{eff}$ is the effective threshold such that $\sqrt{s_\mathrm{eff}}$ lies between the mass of the ground-state and the first excited state [1], namely $s_\mathrm{eff}=(M_H + z_\mathrm{eff})^2$ with $z_\mathrm{eff} \simeq$ 0.4–0.5 GeV.

Nonperturbative effects appear on the r.h.s. of (1) at two places: as power corrections given in terms of vacuum condensates and in the effective threshold $s^{(N)}_\mathrm{eff}$. Depending on the chosen value of *N*, nonperturbative effects are distributed in a different way between power corrections and the effective threshold. Perturbative effects are encoded in the spectral density $\rho _\mathrm{pert}$, in the effective threshold $s^{(N)}_\mathrm{eff}$ and in the power corrections $\varPi ^{(N)}_\mathrm{power}$.

Recall that Eq. (1) is based on modelling the hadron continuum as the effective continuum, i.e., on the substitution $\rho _\mathrm{cont}(s) = \theta (s - s_\mathrm{eff})\rho _\mathrm{pert}(s)$. This relation is fulfilled point-wise at large values of *s* above some $s_\mathrm{up}$, but is a “weak” relation and requires an appropriate smearing for *s* in the mid-energy region above the physical hadron continuum threshold $s_\mathrm{th}$. An appropriate smearing is reached by performing the Borel transform

2 $$\begin{aligned} \int _{s_\mathrm{th}}^{s_\mathrm{up}} \mathrm{d}s \rho _\mathrm{hadr}(s) \mathrm{e}^{-s \tau } = \int _{s_\mathrm{eff}}^{s_\mathrm{up}} \mathrm{d}s \rho _\mathrm{pQCD}(s) \mathrm{e}^{-s \tau } . \end{aligned}$$

For nonzero $\tau$, the contribution of the hadron continuum given by (2) is exponentially suppressed compared to the ground-state contribution (1). Therefore, in the conventional use of QCD sum rules one works in some window of nonzero values of $\tau$. However, one may ask whether or not Eq. (1) may be extended down to $\tau =0$, the so-called local-duality (LD) limit.^1^ Obviously, at $\tau =0$ an appropriate smearing in (2) is guaranteed by the integration; on the other hand, the excited states are not suppressed and one can doubt that modelling the hadron continuum as the effective continuum remains a good approximation at small $\tau$.

First, note that power corrections contain singular terms of the form $\tau ^{2-N}\log (\tau )$. Therefore, the limit $\tau \rightarrow 0$ cannot easily be taken in the sum rule (1) for $N\ge 2$. For $N=0$ and $N=1$, the limit $\tau \rightarrow 0$ in (1) is mathematically well defined. To demonstrate that this limit is also physically meaningful, one needs to show that the corresponding $s_\mathrm{eff}$ indeed lies in the expected range.

**Fig. 1:**
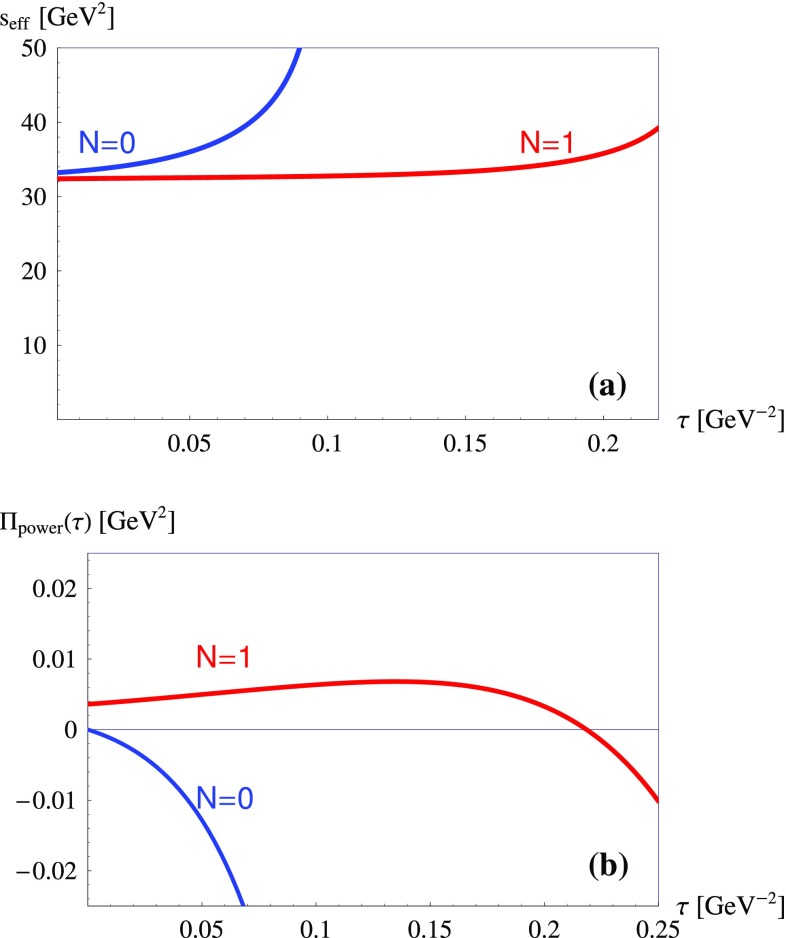
**a** The effective threshold obtained by solving Eq. (1) in the case of the $B^*$-meson for $N=0$ and $N=1$, using in the r.h.s. the leading order (LO), NLO and NNLO perturbative contributions and the condensates up to dimension 6, while the l.h.s. is calculated adopting the known values for $f_{B^*}$ [30] and $M_{B^*}$ [31]. **b** Truncated power corrections including condensates up to dimension 6 for $N=0$ (see Sect. 2) and for $N=1$ [32–34]

Figure 1a presents the effective threshold obtained in the case of the vector $B^*$-meson by solving Eq. (1) for $N=0$ and $N=1$, using in its l.h.s. the results of recent lattice QCD simulations for $f_{B^*}$ [30] and the experimental value for $M_{B^*}$ [31]. Figure 1b shows the truncated series of power corrections including operators of dimension up to 6 for both $N=0$ and $N=1$. Note that power corrections at $\tau =0$ vanish for $N=0$ and take a finite value for $N=1$. The vanishing of $N=0$ power corrections at $\tau =0$ is related to the absence in QCD of a dimension-2 condensate. Obviously, the truncated power corrections for $N=1$ remain under control in a rather broad range of $\tau$, but for $N=0$ explode relatively soon as $\tau$ increases.

It is clear that for the $N=1$ case the OPE is under good control in a broad range of $\tau$ and therefore the lower boundary of the Borel window can be safely extended down to $\tau =0$. For $N=0$ only at relatively small $\tau$ the OPE is under control and the approximation of a $\tau$-independent effective threshold may work well. The relevance of the unknown higher-order power corrections is reflected by the sharp rise of the effective threshold visible in Fig. 1a. Obviously, the standard QCD sum-rule analysis based on a stability window with a constant effective threshold may be problematic. In this respect an alternative approach based on a $\tau$-dependent effective threshold seems to be more appropriate, but this issue goes well beyond the scope of the present paper. Here, the only important property is that the effective threshold at $\tau =0$ has the value expected on the basis of the standard considerations [1], i.e., it is around $(M_{B^*} + z_\mathrm{eff})^2$ with $z_\mathrm{eff} \sim$ 0.4–0.5 GeV. Therefore, for the correlators with $N<2$, modelling the hadron continuum as an effective continuum remains a valid and equally accurate approximation as $\tau \rightarrow 0$, which does not represent a point of discontinuity.

In this work we show that the sum rule (1) for $N=0$ can be of particular interest. Obviously, considering the sum rule at only one point, $\tau =0$, does not allow for the use of the usual sum-rule stability criteria [1] for determining $s_\mathrm{eff}$. Consequently, the decay constants cannot be determined entirely within the QCD sum rules; some “external” inputs are needed to determine $s_\mathrm{eff}$. Nevertheless, in this work we will show that, besides the reduction of the uncertainties related to the absence of the condensates, the LD sum rules represent an efficient tool to investigate the dependence of the pseudoscalar and vector meson decay constants on quark masses and their perturbative behaviour in QCD. Moreover, the LD sum rules turn out to be particularly suitable for the analysis of the strong IB effects in the two-point functions when implemented with only few “external” inputs, e.g., from experiment or lattice QCD.

### Strong isospin breaking from a QCD sum rule in the LD limit

We are interested in the dependence of the decay constants of heavy–light mesons on the quark masses, in particular, in the strong IB effects in the decay constants (i.e., the difference between the decay constants of $\bar{Q}d$ and $\bar{Q}u$ mesons induced by the small mass difference $\delta m=m_d-m_u$). We therefore need to properly take into account all effects depending on the light-quark flavour *q* in the correlation function of the appropriate $\bar{Q} q$ interpolating currents.

Clearly, the $m_q$-dependence on the l.h.s. of (1) is encoded both in the decay constant $f_H$ and in the meson mass $M_H$. On the r.h.s, the IB effects come from several sources: the $m_q$-dependence of $\rho _\mathrm{pert}(s,m_Q,m_q,\alpha _s)$, the $m_q$-dependence of the effective threshold $s_\mathrm{eff}$, the $m_q$-dependence of the power corrections, and the flavour dependence of the quark condensates, in particular, of $\langle \bar{q}q\rangle$. In general, all these effects mix together, which renders the goal of isolating the IB effects in $f_H$ a complicated task. A careful analysis has been carried out recently in [21], following the standard choice $N=2$ for pseudoscalar and $N=1$ for vector mesons.

There is, however, a special case which makes the sum rule (1) particularly suitable for the analysis of IB effects. As discussed above, for $N=0$ and $N=1$ power corrections are regular functions at $\tau =0$. Moreover, for $N=0$ power corrections at $\tau =0$ vanish (power corrections for $N=1$ are nonzero at $\tau =0$) and Eq. (1) is reduced to

3 $$\begin{aligned} f_H^2=\int \limits _{(m_Q+m_q)^2}^{s_\mathrm{eff}(m_Q,m_q,\alpha _s)} \mathrm{d}s \rho _\mathrm{pert}(s,m_Q,m_q,\alpha _s). \end{aligned}$$

On the l.h.s., the $M_H$ contribution has dropped out, thus opening a direct access to the $m_q$ dependence of the decay constant $f_H$. Since power corrections do not contribute to the sum rule in the LD limit, all nonperturbative effects enter now through a single quantity – the effective threshold. The functional dependence of the perturbative spectral density on the quark masses $m_Q$ and $m_q$ can be calculated to the necessary accuracy. The functional dependence of $s_\mathrm{eff}$ on the quark masses may be determined from the general properties of the decay constants of heavy–light mesons in QCD. Namely, its dependence on $m_q$ may be parameterized by a polynomial formula in $m_q$ plus chiral logs, which can be determined by matching to heavy-meson chiral perturbation theory. The numerical coefficients in the polynomial function are not known but may be determined using only few results on the decay constants from lattice QCD, e.g., for nonstrange and strange heavy mesons. Having at our disposal the explicit $m_q$ dependence of the effective threshold and of the spectral densities opens direct access to the strong IB effects related to the small difference of the *u*- and *d*-quark masses in QCD.

We will demonstrate that the main $m_q$ dependence of the decay constants originates from the calculable $m_q$ dependence of the perturbative spectral densities. Therefore, the LD limit opens the possibility of a reliable analysis of the $m_q$ dependence and the strong IB effects in the decay constants of heavy–light mesons (and, in principle, also in other quantities).

This paper is organized as follows: in Sect. 2, we recall the spectral densities of the QCD correlation functions relevant for our LD sum-rule analysis. In Sect. 3, we study the $m_Q$- and $m_q$-dependences of the effective thresholds by making use of an appropriate mass scheme (pole mass and running mass) for the heavy quarks. In Sect. 4, we perform the numerical analysis of the decay constants of heavy–light pseudoscalar and vector mesons and obtain predictions for strong IB effects in the decay constants. Section 5 gives our conclusions. The Appendix collects some details of treating the IB effects within the OPE, which, in our opinion, deserve to be presented.

## Local-duality sum rules for $f_P$ and $f_V$

Let us consider two-point QCD sum-rules for decay constants of pseudoscalar (*P*) and vector (*V*) mesons built up of one massive quark *Q* with mass $m_Q$ and one light quark *q* with mass $m_q$. We consider the axial-vector current

4 $$\begin{aligned} j_\mu ^5(x)=\bar{q}(x) \gamma _\mu \gamma _5 Q(x) \end{aligned}$$

and the vector current

5 $$\begin{aligned} j_\mu (x)=\bar{q}(x) \gamma _\mu Q(x) \end{aligned}$$

as interpolating currents for the pseudoscalar and vector mesons, respectively. The corresponding correlation functions involve two Lorentz structures, the transverse structure $g_{\mu \nu }p^2-p_\mu p_\nu$ and the longitudinal structure $p_\mu p_\nu$:

6 $$\begin{aligned} \varPi ^{5}_{\mu \nu }(p)= & {} i\int \mathrm{d}x \mathrm{e}^{i p x} \langle T( j_\mu ^{5}(x){j^{5}_\nu }^{\dagger }(0))\rangle \nonumber \\= & {} (g_{\mu \nu }p^2-p_\mu p_\nu )\varPi ^{5}_\mathrm{T}(p^2)+p_\mu p_\nu \varPi ^{5}_\mathrm{L}(p^2), \end{aligned}$$

7 $$\begin{aligned} \varPi _{\mu \nu }(p)= & {} i\int \mathrm{d}x \mathrm{e}^{i p x} \langle T( j_\mu (x){j_\nu }^{\dagger }(0))\rangle \nonumber \\= & {} (g_{\mu \nu }p^2-p_\mu p_\nu )\varPi _\mathrm{T}(p^2)+p_\mu p_\nu \varPi _\mathrm{L}(p^2) . \end{aligned}$$

For $\varPi ^5_{\mu \nu }(p)$, we study the longitudinal structure $p_\mu p_\nu$, as it contains the ground-state pseudoscalar-meson contribution

8 $$\begin{aligned} p_\mu p_\nu \frac{f^2_{P}}{M_{P}^2-p^2}, \end{aligned}$$

with

9 $$\begin{aligned} \langle 0|\bar{q} \gamma _\mu \gamma _5 Q|P(p)\rangle =i f_{P}p_\mu . \end{aligned}$$

For $\varPi _{\mu \nu }(p)$, we study the transverse structure $g_{\mu \nu }p^2-p_\mu p_\nu$, which contains the ground-state vector-meson contribution

10 $$\begin{aligned} (g_{\mu \nu }M_V^2-p_\mu p_\nu )\frac{f^2_{V}}{M_{V}^2-p^2}, \end{aligned}$$

with

11 $$\begin{aligned} \langle 0|\bar{q} \gamma _\mu Q|V(p)\rangle =M_{V}f_{V}\epsilon _\mu (p). \end{aligned}$$

As already noted, power corrections for dimension-2 correlation functions vanish in the LD limit $\tau =0$.

We now present their explicit form. The leading power correction to $\varPi ^5_{\mu \nu }(p)$ is given by the quark condensate and easily derived:

12 $$\begin{aligned} \varPi ^5_{\mu \nu }(p)|_{\langle \bar{q}q\rangle }= & {} - (p^2 g_{\mu \nu }-p_\mu p_\nu )m_Q\langle \bar{q}q\rangle \nonumber \\&\times \left[ \frac{1}{p^2}\left( \frac{1}{m_Q^2-p^2}+\frac{\frac{1}{2} m_Q m}{(m_Q^2-p^2)^2}\right) \right] \nonumber \\&+p_\mu p_\nu \langle \bar{q}q\rangle \left[ \frac{-m_Q}{p^2}\left( \frac{1}{m_Q^2-p^2}+\frac{\frac{1}{2} m_Q m}{(m_Q^2-p^2)^2}\right) \right. \nonumber \\&+\left. \frac{m}{(m_Q^2-p^2)^2}\right] . \end{aligned}$$

The Borel transform $p^2\rightarrow \tau$ [defined such that $\frac{1}{a-p^2}\rightarrow \mathrm{e}^{-a \tau }$] of $\varPi ^5_{\mu \nu }(p,q=0)|_{\langle \bar{q}q\rangle }$ reads

13 $$\begin{aligned}&- (p^2 g_{\mu \nu }-p_\mu p_\nu )\frac{\langle \bar{q}q\rangle }{m_Q} \bigg [1-\mathrm{e}^{-m_Q^2\tau } \nonumber \\&\quad +\,\, \frac{m}{2m_Q} (1-\mathrm{e}^{-m_Q^2\tau }-\tau m_Q^2 \mathrm{e}^{-m_Q^2\tau } )\bigg ]\nonumber \\&\quad +\,\, p_\mu p_\nu \frac{\langle \bar{q}q\rangle }{2m_Q^2} [-(2m_Q+m) (\mathrm{e}^{-m_Q^2\tau }-1 ) \nonumber \\&\quad +\,\, m\, m_Q^2\tau \mathrm{e}^{-m_Q^2\tau }]. \end{aligned}$$

By changing the sign of the light-quark mass, the power corrections for the vector correlator $\varPi _{\mu \nu }(p,m_Q,m_q)$ are easily found from $\varPi ^5_{\mu \nu }(p,m_Q,m_q)$: $\varPi ^\mathrm{power}_{\mu \nu }(p,m_Q,m_q)=\varPi ^\mathrm{5,power}_{\mu \nu }(p,m_Q,-m_q)$. Obviously, the Borelized power corrections to both the pseudoscalar and the vector correlators vanish in the limit $\tau =0$.

Since the power corrections do not contribute to the LD sum rule under consideration, we need to consider only the perturbative contributions. After applying the duality cuts at $s_\mathrm{eff}$, separately in the pseudoscalar and the vector channels, performing the Borel transform $p^2\rightarrow \tau$, and setting $\tau \rightarrow 0$, the corresponding sum rules take the form

14 $$\begin{aligned} f_{P,V}^2=\int \limits _{(m_Q+m_q)^2}^{s_\mathrm{eff}}\mathrm{d}s \rho ^\mathrm{pert}_{P,V}(s,m_Q,m_q). \end{aligned}$$

The functions $\rho ^\mathrm{pert}_{P}$ and $\rho ^\mathrm{pert}_{V}$ in (14) are the spectral densities of the invariant functions $\varPi ^5_{L}(p^2)$ and $\varPi _{T}(p^2)$, respectively.

Let us emphasize that in (14) both the full spectral densities and the decay constants are scale independent quantities. Therefore, the effective thresholds are scale independent objects, too. In perturbation theory, the spectral densities are calculated as power expansions in $a \equiv \alpha _s(\mu )/\pi$, $\alpha _s(\mu )$ the strong coupling in the $\overline{\mathrm{MS}}$-scheme at scale $\mu$:

15 $$\begin{aligned} \rho ^\mathrm{pert}_{i}(s,m_Q,m_q)= & {} \rho _{i}^{(0)}(s,m_Q,m_q)\nonumber \\&+\,\, a\rho _{i}^{(1)}(s,m_Q,m_q) \nonumber \\&+\,\, a^2 \rho _{i}^{(2)}(s,m_Q,m_q) \nonumber \\&+\, O(a^3) \end{aligned}$$

with $i=P,V$. In practice, one adopts truncated expansions of the spectral densities; this leads to a scale dependence of the spectral densities. As the result, the effective thresholds will also depend on the scale, to compensate the scale dependence of the spectral densities emerging in the course of truncation. Explicitly, the leading-order (LO) spectral densities read

16 $$\begin{aligned} \rho _P(s,m_Q,m)= & {} \frac{N_c}{8\pi ^2} (s-(m_Q-m_q)^2 ) \nonumber \\&\times (m_Q+m_q)^2\frac{\lambda ^{1/2}(s,m_Q^2,m_q^2)}{s^3} \nonumber \\&\times \theta (s-(m_Q+m_q)^2), \end{aligned}$$

17 $$\begin{aligned} \rho _V(s,m_Q,m)= & {} \frac{N_c}{24\pi ^2} (s-(m_Q-m_q)^2 ) \nonumber \\&\times \left( 2s+(m_Q+m_q)^2\right) \frac{\lambda ^{1/2}(s,m_Q^2,m_q^2)}{s^3} \nonumber \\&\times \theta (s-(m_Q+m_q)^2). \end{aligned}$$

Obviously, the lower integration limit in (14) is determined by the threshold in the correlation functions.

In Eq. (15), we may employ different definitions of the quark masses: The most advanced calculation of the pseudoscalar and vector spectral densities including order-$O(a^2)$ terms was performed [35, 36], for a massless light quark, in terms of the heavy-quark pole mass. The expansion in terms of the heavy-quark pole mass is appropriate for considering the heavy-quark limit, which we address in Sect. 3.1.

However, the pole-mass expansion leads to a rather slow convergence of the perturbative expansion for the decay constants [15–18, 37]. The convergence improves considerably when one rearranges the perturbative expansion in terms of the running $\overline{\mathrm{MS}}$ masses. Therefore, for the practical analysis of the $m_q$-dependences of the meson decay constants in Sect. 4, we make use of the perturbative expansion in terms of the running $\overline{\mathrm{MS}}$ masses of the light and the heavy quarks. The corresponding NLO and NNLO functions $\rho _{P}^{(i)}$ ($i=1,2$) in (15) necessary for such an analysis are found from the spectral densities of the pseudoscalar correlation function given in [37] by multiplying them by $1/s^2$. Similarly, the transverse spectral densities $\rho _{V}^{(i)}$ in (15) are found from the spectral densities of [32–34] by multiplying them by 1/*s*. In our analysis, we make use of the exact LO perturbative spectral density given by (16), at the NLO we keep the terms $O(a m_q^0)$ and $O(a m_q^1)$, and in the NNLO we keep only the known terms of order $O(a^2m_q^0)$.

We would like to emphasize that the perturbative spectral density (15) does not generate terms of order $m_q\log (m_q)$ in the dual correlator (14). This observation will be crucial for discussing properties of the effective thresholds in the next section.

## Dependence of the effective thresholds on the quark masses

Let us now consider the dependences of the effective threshold on the quark masses $m_Q$ and $m_q$.

### Heavy-quark limit in the pole-mass scheme

We start with the heavy-quark limit of the decay constants, originally discussed in Refs. [38, 39] within the Heavy Quark Effective Theory (HQET). In what follows, however, we do not consider the static decay constants and we work in full QCD.

For the sake of argument we consider first a massless light quark: $m_q=0$. We can make use of any scheme for the heavy-quark mass, but we start with the pole-mass scheme, which leads to a more transparent behaviour of the effective threshold. We first isolate the pole mass, which we denote $M_Q$, in the effective threshold:

18 $$\begin{aligned} \sqrt{s_\mathrm{eff}}=M_Q+z^\mathrm{pole}_\mathrm{eff}(M_Q). \end{aligned}$$

For the decay constant of pseudoscalar and vector $\bar{Q}q$ mesons, using results from [35, 36], we obtain in the limit $M_Q\rightarrow \infty$

19 $$\begin{aligned} f_{P}^2M_Q= & {} \frac{N_c}{3\pi ^2}(z^\mathrm{pole}_\mathrm{eff})^3\bigg [1+\bar{a}\,\frac{C_F}{12} \{45+4\pi ^2 \nonumber \\&+ 18\log (M_Q/2 z^\mathrm{pole}_\mathrm{eff} ) \}+O(\bar{a}^2)\bigg ], \nonumber \\ f_{V}^2M_Q= & {} \frac{N_c}{3\pi ^2}(z^\mathrm{pole}_\mathrm{eff})^3\bigg [1+\bar{a}\,\frac{C_F}{12} \{33+4\pi ^2 \nonumber \\&+ 18\log (M_Q/2 z^\mathrm{pole}_\mathrm{eff})\}+O(\bar{a}^2)\bigg ]. \end{aligned}$$

Hereafter, we denote $\bar{a}\equiv \bar{\alpha }_s(M_Q)/\pi$, $\bar{\alpha }_s(M_Q)$ the running strong coupling in the $\overline{\mathrm{MS}}$ scheme at scale $M_Q$, and we use the standard notations $C_F=(N_c^2-1)/(2N_c)$, $C_A=N_c$, $T=1/2$, and $n_l$ the number of massless quarks [35, 36].

Since only the near-threshold behaviour of the spectral densities is relevant for the leading behaviour in the large-$M_Q$ limit, we may obtain also the $O(\bar{a}^2)$ terms in the dual correlation functions [i.e., the r.h.s. of (19)] from the analytical expressions for these spectral densities given by Eqs. (30) and (31) of [35, 36].

In the limit $M_Q\rightarrow \infty$, the dual correlation functions, expressed in terms of $z^\mathrm{pole}_\mathrm{eff}(M_Q)$, do not contain corrections of order $\bar{a}^n M_Q$ (this property will not hold in the running-mass scheme) but still contain $\log (M_Q)$ terms of the type $(\bar{a} \log (M_Q/z^\mathrm{pole}_\mathrm{eff} ) )^n$, $\bar{a} (\bar{a} \log (M_Q/z^\mathrm{pole}_\mathrm{eff} ) )^{n-1}$, etc. The terms $(\bar{a} \log (M_Q/z^\mathrm{pole}_\mathrm{eff} ) )^n$, although formally of order $\bar{a}^n$, remain unsuppressed in the limit $M_Q\rightarrow \infty$. To treat all terms containing $\log (M_Q)$, it is important to emphasize that they are exactly the same in the vector and the pseudoscalar sum rules. Therefore, they may be resummed by introducing a properly defined effective threshold $z^\mathrm{HQ}_\mathrm{eff}$, one and the same in the pseudoscalar and the vector channels. The explicit relation between $z^\mathrm{pole}_\mathrm{eff}(M_Q)$ and $z^\mathrm{HQ}_\mathrm{eff}$, including also the $a^2$ terms, reads

20 $$\begin{aligned} z^\mathrm{pole}_\mathrm{eff}(M_Q)= & {} z^\mathrm{HQ}_\mathrm{eff} [ 1-\bar{a}\, d_{11}\log ({M_Q}/{z^\mathrm{HQ}_\mathrm{eff}}) \nonumber \\&- \bar{a}^2 d_{22} (\log ({M_Q}/{z^\mathrm{HQ}_\mathrm{eff}} ) )^2 \nonumber \\&- \bar{a}^2 d_{21}\log ({M_Q}/{z^\mathrm{HQ}_\mathrm{eff}} )+O(\bar{a}^3) ],\nonumber \\ d_{11}= & {} \frac{C_F}{2}, \nonumber \\ d_{22}= & {} \frac{C_F}{24} [11 C_A-3 C_F-4 n_l T ],\nonumber \\ d_{21}= & {} \frac{C_F}{432} [C_F(63+48\pi ^2) \nonumber \\&+ C_A(1401+76\pi ^2-396\log 2) \nonumber \\&- 4 n_l T(129+8\pi ^2-36\log 2) ]. \end{aligned}$$

The new quantity $z^\mathrm{HQ}_\mathrm{eff}$, which has the meaning of the effective threshold in HQET, absorbs all $\log (M_Q)$ terms on the r.h.s. of the sum rules (19); the latter assume a form in which the HQ limit may easily be taken:

21 $$\begin{aligned} f_{P}^2M_Q= & {} \frac{N_c}{3\pi ^2}(z^\mathrm{HQ}_\mathrm{eff})^3 \bigg [1+ \bar{a} \frac{C_F}{12} \{45+4\pi ^2-18\log 2 \} \nonumber \\&+ O(\bar{a}^2)\bigg ],\nonumber \\ f_{V}^2M_Q= & {} \frac{N_c}{3\pi ^2}(z^\mathrm{HQ}_\mathrm{eff})^3 \bigg [1+ \bar{a} \frac{C_F}{12} \{33+4\pi ^2-18\log 2 \} \nonumber \\&+ O(\bar{a}^2)\bigg ]. \end{aligned}$$

We did also calculate the $O(\bar{a}^2)$ contributions but do not present their explicit expressions here. The expressions (21) immediately lead to the ratio of the decay constants in the heavy-quark limit [40, 41]. Including also $O(\bar{a}^2)$ corrections, we obtain^2^

22 $$\begin{aligned} \frac{f_{V}}{f_P}= & {} 1-\bar{a}\frac{C_F}{2}+\bar{a}^2\frac{C_F}{144} \{ 93 C_F +4(-41+19 n_l)T \nonumber \\&+C_A (-263-24\pi ^2(\log 2-1) ) \nonumber \\&+16\pi ^2 (T+C_F(\log 8 -4) ) \nonumber \\&+36(C_A-2 C_F)\zeta _3 \} \nonumber \\&+O(\bar{a}^3), \end{aligned}$$

with $\zeta _3\simeq 1.202$. The $O(\bar{a}^2)$ term in (22) reproduces the result first presented in Eq (3.12) of [41].

Notice that for finite $M_Q$, $z^\mathrm{pole}_\mathrm{eff}$ contains not only the logarithmic corrections, which are the same in the pseudoscalar and the vector channels, but also the $1/M_Q$ corrections,

23 $$\begin{aligned} z^\mathrm{pole}_\mathrm{eff}= & {} z^\mathrm{HQ}_\mathrm{eff} \bigg [1-\bar{a}\frac{C_F}{2} \log ({M_Q}/{z^\mathrm{HQ}_\mathrm{eff}} ) \nonumber \\&+O(\bar{a}^2) \bigg ]+O(1/M_Q), \end{aligned}$$

which are different for the thresholds in the pseudoscalar and the vector sum rules.

### Combined heavy-quark and chiral limits in the pole-mass scheme

The results (19) are obtained for a massless light quark. Switching on a small light-quark mass $m_q$, the leading corrections generated by integration of the perturbative spectral densities are proportional to $m_q$. As already noted in [21], no chiral logs of the kind $m_q \log (m_q)$ arise from integrating the spectral densities. Therefore, chiral logs in the decay constants may be generated only by chiral logs in the effective threshold. Moreover, in order to study the chiral logs in the decay constants, it is sufficient to make use of the perturbative spectral densities for $m_q=0$. On the other hand, heavy-meson chiral perturbation theory (HMChPT) (S. R. Sharpe, private communication; see the appendix in [21]) [42], requires the appearance of chiral logs, which we denote as $z^\mathrm{HQ}_L$ in the chiral expansion of the decay constants in the heavy-quark limit. Since the only source of such terms is the effective threshold, we write

24 $$\begin{aligned} z^\mathrm{HQ}_\mathrm{eff}=z^\mathrm{HQ}_{0} (1+z^\mathrm{HQ}_{L} )+\cdots , \end{aligned}$$

where the dots denote linear and higher-order terms in the light-quark mass $m_q$. The coefficient $z^\mathrm{HQ}_{L}$ can now be fixed by matching to HMChPT (S. R. Sharpe, private communication; see the appendix in [21]) [42], which provides the explicit chiral logs $R_\chi (m_q)$ in the ratio $f_{H_q}(m_q)/f_{H_{ud}}$, with $H_{ud}$ a heavy meson with a light valence quark of the average mass $m_{ud}\equiv (m_u+m_d)/2$. Finally, we obtain

25 $$\begin{aligned} z^\mathrm{HQ}_L(m_q)= [1+R_\chi (m_q) ]^{2/3}-1\approx \frac{2}{3} R_\chi (m_q). \end{aligned}$$

The explicit expression for $R_\chi (m_q)$ was derived in (S. R. Sharpe, private communication; see the appendix in [21]) [42] and presented by Eq. (A.3) of [21].

### Quark-mass dependences of the effective threshold in the running-mass scheme

For practical sum-rule analyses of decay constants, one prefers the $\overline{\mathrm{MS}}$ running-mass scheme since it entails a better convergence of the perturbative expansion [15–18, 37]. It is not difficult to perform the limit $\overline{m}_Q(\mu )\rightarrow \infty$ also for the running-mass correlation function. Also therein one can write

26 $$\begin{aligned} \sqrt{s_\mathrm{eff}}=\overline{m}_Q(\mu )+\bar{z}_\mathrm{eff}(\mu ). \end{aligned}$$

The effective threshold $\bar{z}_\mathrm{eff}(\mu )$ in the $\overline{\mathrm{MS}}$ scheme is related to $z^\mathrm{pole}_\mathrm{eff}$ introduced in the pole-mass scheme through an obvious relation which just expresses the fact that the upper integration limit $s_\mathrm{eff}$ is a scheme-independent quantity:

27 $$\begin{aligned} M_Q+z^\mathrm{pole}_\mathrm{eff}=\overline{m}_Q(\mu )+\bar{z}_\mathrm{eff}(\mu ). \end{aligned}$$

In particular, for $\mu =\overline{m}_Q$, taking into account that

28 $$\begin{aligned} M_Q=\overline{m}_Q\left( 1+C_F \bar{a}\right) , \quad \overline{m}_Q\equiv \overline{m}_Q(\overline{m}_Q), \end{aligned}$$

one finds

29 $$\begin{aligned} \bar{z}_\mathrm{eff}(\overline{m}_Q)=z^\mathrm{pole}_\mathrm{eff}+C_F \bar{a} \overline{m}_Q+O(\bar{a}^2). \end{aligned}$$

Since $z^\mathrm{pole}_\mathrm{eff}$ does not contain terms scaling as $M_Q$ in the limit $M_Q\rightarrow \infty$, $\bar{z}_\mathrm{eff}(\mu )$ should contain terms which diverge as powers of $a^n M_Q$ in this limit. This is, of course, no obstacle for using $\bar{z}_\mathrm{eff}(\mu )$ in the analysis of the decay constants of charmed or beauty mesons but makes this quantity not particularly convenient for studying the heavy-quark limit of the sum rules. The terms in $\bar{z}_\mathrm{eff}(\mu )$ divergent as $m_Q\rightarrow \infty$, however, do not lead to divergent terms in the decay constants; also, the behaviour of the spectral densities in the $\overline{\mathrm{MS}}$ scheme is a bit more tricky than in the pole-mass scheme. The dual correlator is determined by the end-point behaviour of the spectral densities; as already mentioned in [37], the higher-order spectral densities in the $\overline{\mathrm{MS}}$ scheme do not vanish at the threshold but behave as $v^{2-k}\alpha _s\log (v)^k$, $v=\frac{1-s/M_Q^2}{1+s/M_Q^2}$. Finally, when the $\overline{\mathrm{MS}}$ spectral densities are used and the duality cut is expressed via $z^\mathrm{HQ}_\mathrm{eff}$, all terms containing powers of $\overline{m}_Q$ – those coming from the integrals of the spectral densities and those contained in $z_\mathrm{eff}(\overline{m}_Q)$ – cancel each other, yielding a sum rule for $f_H^2$ which can also be obtained just by expressing $M_Q$ via $\overline{m}_Q$ in (19), e.g.,

30 $$\begin{aligned} f_{P}^2\overline{m}_Q= \frac{N_c}{3\pi ^2}\left( z^\mathrm{HQ}_\mathrm{eff}\right) ^3\left[ 1+\frac{1}{12}\bar{a} C_F(33+4\pi ^2-18 \log 2)\right] .\nonumber \\ \end{aligned}$$

Let us now switch on a small light-quark mass $m_q$. The spectral densities are now treated as functions of $\overline{m}_Q(\mu )$ and $\overline{m}_q(\mu )$. Taking into account that the effective threshold depends on the scale $\mu$ only because of the truncation of the perturbative series, and that the chiral logs have been fixed in the pole-mass scheme, it is convenient to work with the following parameterisation for $s_\mathrm{eff}$:

31 $$\begin{aligned} \sqrt{s_\mathrm{eff}}= & {} M^{(2)}_Q+z_\mathrm{eff}^\mathrm{pole}(1+z_L^\mathrm{HQ})+\overline{m}_q(\mu )+\bar{z}'_1(\mu )\overline{m}_q(\mu ) \nonumber \\&+O(\overline{m}_q^2). \end{aligned}$$

The pole mass $M^{(2)}_Q$ here is understood as being expressed via the running mass $\overline{m}_Q(\mu )$ (e.g., [43]) at $O(a^2)$ accuracy, the available accuracy of the correlation function. We can rewrite this expression in a form similar to (26) in terms of $\bar{z}_{0}(\mu )=z_\mathrm{eff}^\mathrm{pole}+\delta \overline{m}_Q(\mu )$, where $\delta \overline{m}_Q(\mu )\equiv M^{(2)}_Q-\overline{m}_Q(\mu )$:

32 $$\begin{aligned} \sqrt{s_\mathrm{eff}}= & {} \overline{m}_Q(\mu )+\overline{m}_q(\mu )+\bar{z}_{0}(\mu ) \nonumber \\&\times \bigg (1+\frac{\bar{z}_{0}(\mu )-\delta \overline{m}_Q(\mu )}{\bar{z}_{0}(\mu )}z_L^\mathrm{HQ} \nonumber \\&+\bar{z}_1(\mu )\overline{m}_q(\mu )\bigg )+O(\overline{m}_q^2). \end{aligned}$$

Let us recall that the chiral logs $z_L^\mathrm{HQ}$ have been calculated in the heavy-quark limit; at finite values of $m_Q$, chiral logs receive corrections which are unknown. So we take into account only the known leading effect of chiral logs, to study whether or not their impact on the IB is crucial. Two other parameters of the effective threshold – $z_\mathrm{eff}^\mathrm{pole}$ and $\bar{z}'_1(\mu )$ if one makes use of the parameterisation (31), or $\bar{z}_{0}(\mu )$ and $\bar{z}_1(\mu )$ if one works with (32) – are unknown and will be fixed by using some external benchmark results for the decay constants from lattice QCD. The inclusion of higher-order terms in the light-quark mass has no impact on the decay constants; thus, such terms are not considered.

## Numerical analysis of the sum rules

Now, we turn to the numerical estimates. For the relevant OPE parameters, we adopt the following numerical input:

33 $$\begin{aligned}&(\overline{m}_d - \overline{m}_u) (2\;\mathrm{GeV}) = (2.67 \pm 0.22)\;\mathrm{MeV} ~[24] , \nonumber \\&\overline{m}_{ud}(2\;\mathrm{GeV}) \equiv \frac{\overline{m}_u + \overline{m}_d}{2} = (3.70 \pm 0.17)\;\mathrm{MeV} ~[24] ,\;\nonumber \\&\overline{m}_s(2\;\mathrm{GeV}) = (93.9 \pm 1.1)\;\mathrm{MeV} ~[24] ,\nonumber \\&\overline{m}_c(\overline{m}_c) = (1.275 \pm 0.025)\;\mathrm{GeV} ~ [14], \nonumber \\&\overline{m}_b(\overline{m}_b) = (4.247 \pm 0.034)\ \mathrm{GeV} ~ [7],\nonumber \\&\alpha _\mathrm{s}(M_Z) = 0.1182 \pm 0.0012 ~ [24]. \end{aligned}$$

We have checked that employing slightly different values of the quark masses [compatible within uncertainties with those in Eq. (33)], which have been reported in the lattice analyses of pseudoscalar mesons ($\overline{m}_b(\overline{m}_b) = (4.190 \pm 0.021)$ GeV, $\overline{m}_c(\overline{m}_c) = (1.286 \pm 0.030)$ [44, 45]) or vector mesons ($\overline{m}_b(\overline{m}_b) = (4.26 \pm 0.10)\;\mathrm{GeV}$ [46], $\overline{m}_c(\overline{m}_c) = (1.348 \pm 0.046)\;\mathrm{GeV}$ [47], $\overline{m}_s(2 \ \hbox {GeV}) = (99.6 \pm 4.3)\;\mathrm{MeV}$ [47]), does not affect our numerical estimates for the IB within the quoted uncertainties.

**Table 1:** Parameters of the effective thresholds and resulting IB in the decay constants of heavy pseudoscalar and vector mesons. The parameter $z_L$ in the effective threshold for the “linear + log” ansatz is fixed by ChHQET in the heavy-quark limit

Meson	Threshold	$z_0\;(\mathrm{GeV})$	$z_1\,[\mathrm{GeV}^{-1}]\quad$	$f_{M_d}-f_{M_u} \ (\mathrm{MeV})$
*D*	Constant	$1.363\pm 0.213$		$1.222\pm 0.219$
	Linear	$1.366\pm 0.203$	$- \ 0.365\pm 0.301$	$1.050\pm 0.102$
	Linear + log	$1.225\pm 0.194$	$- \ 1.422\pm 0.304$	$0.929\pm 0.088$
$D^*$	Constant	$1.207\pm 0.147$		$1.276\pm 0.217$
	Linear	$1.207\pm 0.138$	$0.006\pm 0.464$	$1.281\pm 0.389$
	Linear + log	$1.087\pm 0.139$	$- \ 0.978\pm 0.524$	$1.080\pm 0.381$
*B*	Constant	$1.501\pm 0.143$		$0.792\pm 0.081$
	Linear	$1.499\pm 0.134$	$\;\,0.498\pm 0.076$	$1.113\pm 0.108$
	Linear + log	$1.365\pm 0.136$	$- \ 0.639\pm 0.147$	$0.918\pm 0.091$
$B^*$	Constant	$1.534\pm 0.163$		$0.839\pm 0.076$
	Linear	$1.533\pm 0.152$	$0.227 \pm 0.401$	$1.010\pm 0.317$
	Linear + log	$1.395\pm 0.152$	$- \ 0.938\pm 0.448$	$0.786\pm 0.311$

We work with the effective threshold in the form (32) and consider the following three Ansätze:

1. “Constant” threshold: the $\bar{z}_1(\mu )$ term in the effective threshold (32) and the chiral logs $z_L^\mathrm{HQ}$ are neglected; the only unknown parameter $\bar{z}_{0}(\mu )$ is fixed from the lattice results for the decay constants of the isospin-symmetric heavy mesons, with $m_q=m_{ud}$.
2. “Linear” threshold: the chiral logs $z_L^\mathrm{HQ}$ are neglected and the parameters $\bar{z}_{0}(\mu )$ and $\bar{z}_1(\mu )$ are fixed by the lattice QCD results for the decay constants at two $m_q$ values, for the isospin-symmetric and the strange heavy mesons.
3. “Linear + log” threshold: the known leading chiral logs represented by $z_L^\mathrm{HQ}$ are included; the parameters $\bar{z}_{0}(\mu )$ and $\bar{z}_1(\mu )$ are fixed from the lattice QCD results for the decay constants at two $m_q$ values, for isospin-symmetric and strange heavy mesons.

As we have already noted, because of the truncation of the perturbative expansion, the truncated spectral densities depend on the scale $\mu$. Obviously, the parameters $\bar{z}_0$ and $\bar{z}_1$ are also $\mu$-dependent.

For fixing the parameters of the effective thresholds, we make use of the following results from lattice QCD:

34 $$\begin{aligned}&f_D=(212.15\pm 1.45)\;\mathrm{MeV},\quad \frac{f_{D_s}}{f_D}=1.1716\pm 0.0032 \ [24] ,\nonumber \\&f_{D^*}=(223.5\pm 8.3)\;\mathrm{MeV}, \quad \frac{f_{D^*_s}}{f_{D^*}}=1.203\pm 0.054 \ [13] , \nonumber \\&f_B=(186.0\pm 4.0)\;\mathrm{MeV}, \quad \frac{f_{B_s}}{f_B}=1.205\pm 0.007 \ [24] ,\nonumber \\&f_{B^*}=(186.4\pm 7.1)\;\mathrm{MeV}, \quad \frac{f_{B^*_s}}{f_{B^*}}=1.197 \pm 0.055 \ [13].\nonumber \\ \end{aligned}$$

In these formulae, $f_H$ denotes the decay constant of the isospin-averaged heavy–light mesons with the light-quark mass $m_{ud}$, whereas $f_{H_s}$ denotes the decay constant of the heavy strange mesons.

Table 1 summarizes the effective thresholds corresponding to our three Ansätze and presents estimates of the strong IB effect. For our final estimates, we perform a bootstrap analysis of the uncertainties assuming that the OPE parameters in (33) have Gaussian distributions with corresponding Gaussian errors, whereas the scale $\mu$ has a flat distribution in the range $1< \mu \;(\mathrm{GeV}) <3$ for charmed mesons and $3< \mu \;(\mathrm{GeV}) < 6$ for beauty mesons.

**Fig. 2:**
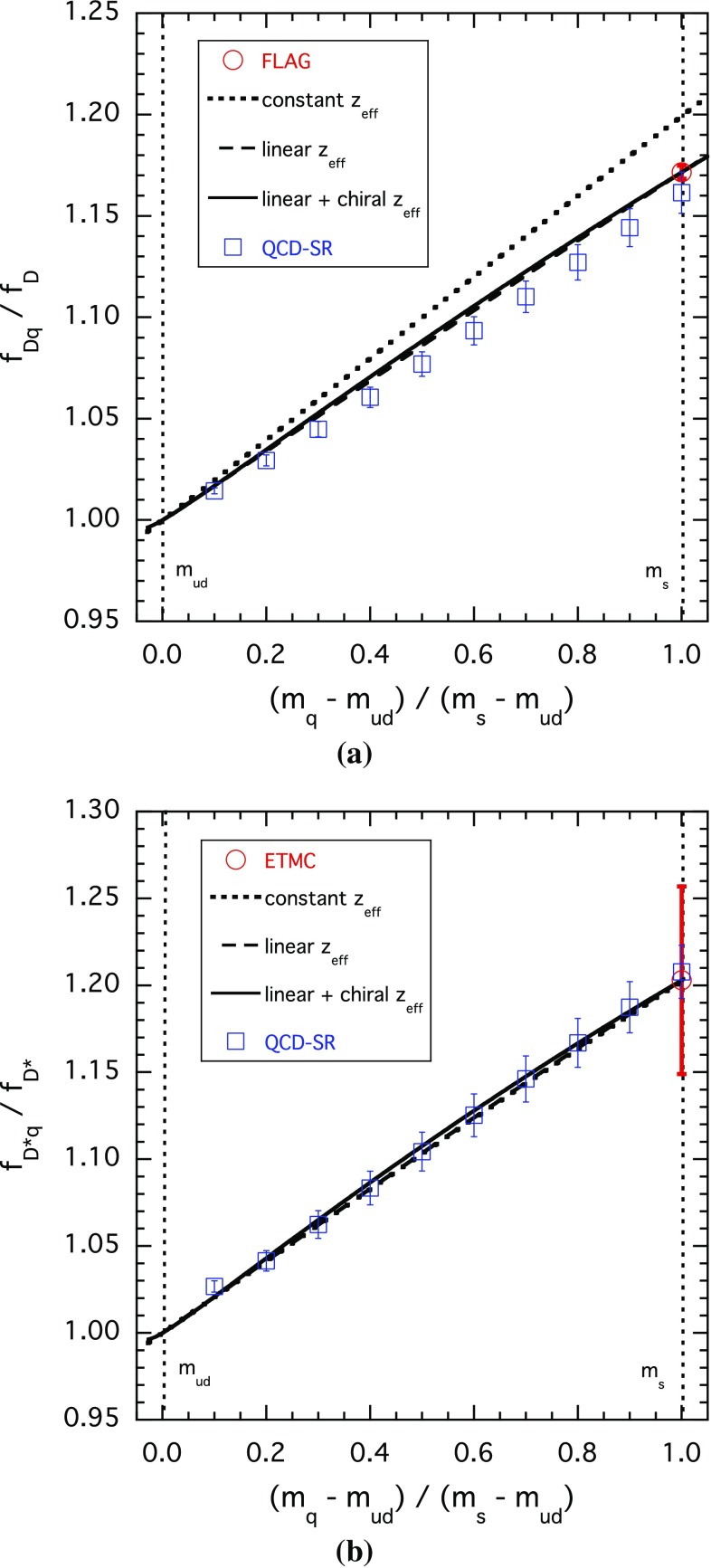
Dependence of the ratio $f(m_q)/f(m_{ud})$ for pseudoscalar $\bar{c} q$ (**a**) and vector $\bar{c} q$ (**b**) mesons. Dotted lines correspond to the constant ($m_q$-independent) effective threshold [ansatz (1)] fixed from the known lattice QCD results for the decay constants of the *D* and $D^*$ mesons. Dashed lines correspond to the effective threshold linear in $m_q$ [ansatz (2)], the parameters of which are fixed by the lattice results for strange heavy mesons $D_s$ and $D^*_s$. Solid lines correspond to the effective threshold containing the known chiral logs in addition to the function linear in $m_q$ [ansatz (3)]. In all cases, the results for the central values of the threshold parameters in Table 1 are displayed. We also show results from an alternative analysis based on Borel QCD sum rules [21]

**Fig. 3:**
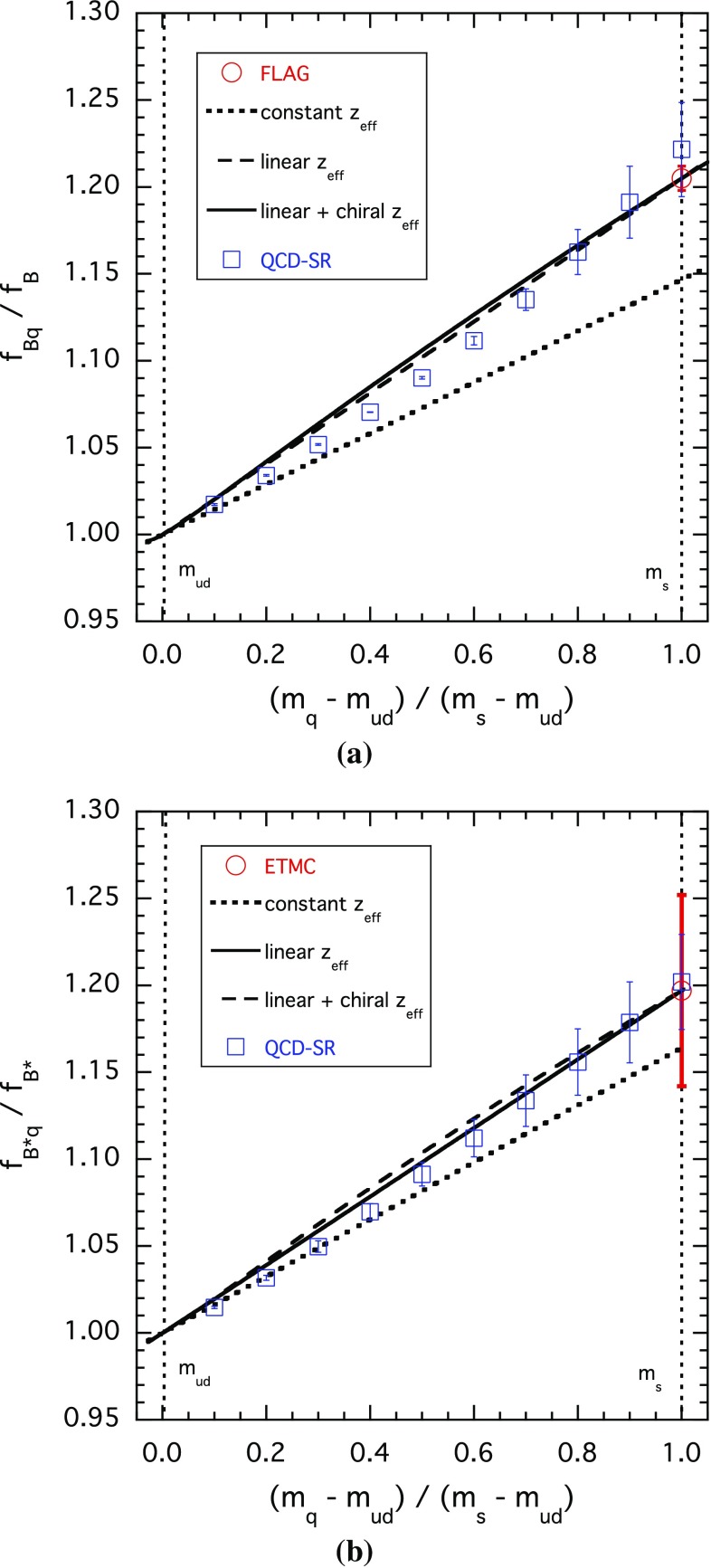
The same as in Fig. 2 but for pseudoscalar $\bar{b} q$ (**a**) and vector $\bar{b} q$ (**b**) mesons

As soon as the effective thresholds are known, we readily get the decay constants $f_{H_q}$ as a function of the scale independent ratio $(\overline{m}_q-\overline{m}_{ud})/(\overline{m}_s-\overline{m}_{ud})$. The results for the ratios of the decay constants $f_{H_q}/f_{H_{ud}}$ are shown in Figs. 2 and 3.

Notice that the results corresponding to a constant effective threshold [ansatz (1)] are quite close to those obtained including the $m_q$-dependence [ansatz (2)] and to the results of Ref. [21], which contain effects in the decay constants at any order in the light-quark mass. So, an important conclusion to be drawn from our results is that effects at order $\mathcal{{O}}(m_q^2)$ in the effective threshold are not crucial for describing the $m_q$-dependence of the decay constants and for estimating the slope of the IB effect at the physical value of the light-quark mass: the latter are both determined to a large extent by the *known* $m_q$-dependence of the spectral densities and can thus be reliably controlled in our approach.

## Summary and conclusions

We addressed the local-duality (LD) limit, $\tau =0$, of the Borel QCD sum rules for the decay constants of heavy–light pseudoscalar and vector mesons. An invaluable feature of the LD limit is that for a proper choice of the correlation function, all vacuum-condensate contributions vanish and the full nonperturbative QCD dynamics is parameterized in terms of merely one quantity – the effective threshold. Our analysis demonstrates that the effective threshold has a nontrivial functional dependence on the masses of the heavy and the light quarks, $m_Q$ and $m_q$, respectively. This dependence has been parameterized in the form suggested by the behaviour of the decay constants in the known limits: the chiral limit for $m_q$ and the heavy-quark limit for $m_Q$. In the heavy-quark limit, we clarify the role played by the radiative corrections in the effective threshold for reproducing the pQCD expansion of the decay constants of pseudoscalar and vector mesons.

This paper elucidates the dependence of the decay constants on a light-quark mass $$m_q$$ in the range $$m_{ud} < m_q<$$$$m_s$$. Fixing a few numerical parameters of the effective threshold by using the available accurate inputs from lattice QCD, we have derived the full analytic dependence of the decay constants $$f_H(m_q)$$ on the light-quark mass $$m_q$$. The resulting dependence of the decay constants $$f_H(m_q)$$ on $$m_q$$ emerges from two sources: (i) from the $$m_q$$-dependence of the QCD perturbative spectral densities known explicitly as expansion in powers of $$\alpha _s$$ and (ii) from the $$m_q$$-dependence of the effective threshold known approximately. An important outcome of our analysis is that the variation of the decay constants with respect to $$m_q$$ comes to a great extent (70–80% of the full effect) comes from the rigorously calculable dependence on $$m_q$$ of the perturbative spectral densities and is therefore under a good theoretical control.

Noteworthy, the known perturbative expansion of the correlation functions [32–37], where the sea-quark mass effects are neglected, limits the accuracy of the decay constants of the heavy–light mesons to $O(m_s \bar{a}^2)$ accuracy, $\bar{a}\sim 0.1$ at the appropriate renormalisation scales. Therefore the accuracy of the individual decay constants obtained from QCD sum rules does not exceed a few MeV. Nevertheless, we would like to emphasize that the IB difference of the decay constants, $f_{M_d}-f_{M_u}$, where the sea-quark contributions of order $O(m_{s,u,d} \bar{a}^2)$ cancel each other, may be predicted with a much higher accuracy, $O(\delta m\bar{a}^2)$. Therefore, the proposed method can potentially provide a higher accuracy of the IB effects than other approaches.

As our final estimates of the IB, we take the average of the results corresponding to the linear and the linear + log effective thresholds in Table 1:

35 $$\begin{aligned} f_{D^+} - f_{D^0}= & {} (0.96 \pm 0.09) \ \mathrm{MeV} , \end{aligned}$$

36 $$\begin{aligned} f_{D^{*+}} - f_{D^{*0}}= & {} (1.18 \pm 0.35) \ \mathrm{MeV} , \end{aligned}$$

37 $$\begin{aligned} f_{B^0} - f_{B^+}= & {} (1.01 \pm 0.10) \ \mathrm{MeV} , \end{aligned}$$

38 $$\begin{aligned} f_{B^{*0}} - f_{B^{*+}}= & {} (0.89 \pm 0.30) \ \mathrm{MeV} . \end{aligned}$$

Sizeably larger uncertainties of the IB in the decay constants of vector mesons compared to pseudoscalar mesons are related to larger uncertainties of the input lattice QCD results for the corresponding ratios $f_{H_s}/f_{H_{ud}}$.

These estimates are in good agreement with the results of our recent analysis within a different version of QCD sum rules – the Borel sum rules with $\tau$-dependent threshold [21]:

39 $$\begin{aligned} f_{D^+} - f_{D^0}= & {} (0.97 \pm 0.13) ~ \mathrm{MeV} ~ , \end{aligned}$$

40 $$\begin{aligned} f_{D^{*+}} - f_{D^{*0}}= & {} (1.73 \pm 0.27) ~ \mathrm{MeV} ~ , \end{aligned}$$

41 $$\begin{aligned} f_{B^0} - f_{B^+}= & {} (0.90 \pm 0.13) ~ \mathrm{MeV} ~ , \end{aligned}$$

42 $$\begin{aligned} f_{B^{*0}} - f_{B^{*+}}= & {} (0.81 \pm 0.11) ~ \mathrm{MeV} ~ . \end{aligned}$$

The only exception is the $D^*$ case, where one observes tension between these two sets of the results; note, however, that also the uncertainties of these predictions are rather large.

Very recently [48] a new precise determination of the strong IB effect in the decay constants of D- and B-mesons has been carried out by the FNAL and MILC lattice collaborations.

In the charm sector their result is $f_{D^+} - f_{D^0} = 1.13 (15)$ MeV, which nicely agrees with our findings (35) and (39). As for the bottom sector, it is shown that the available HPQCD and RBC/UKQCD calculations [49–51] overestimate significantly the strong IB effect because of an inappropriate use of unitary lattice points (i.e. those having the same mass for valence and sea light-quarks). The FNAL/MILC result is $f_{B^0} - f_{B^+} = 1.12 (15)$ MeV, which is in excellent agreement with our findings (37) and (41).

Thus, our sum-rule predictions are nicely confirmed quantitatively by lattice QCD both for the central values and the overall uncertainties. This is reassuring: the strong IB effect and its uncertainty in the decay constants of heavy–light mesons can be reliable and accurately estimated within the QCD sum-rule approach.

It should be emphasized that the present approach based on the combination of OPE and a few inputs from lattice QCD potentially has fewer theoretical uncertainties than other formulations of QCD sum rules: first, the condensate contributions, in particular, those of the quark condensate, which produced the main OPE error in the decay constants, vanish in the LD limit; second, the systematic uncertainty of the sum-rule method is now encoded in only one quantity – the effective threshold, which may be fixed to good accuracy due to the use of the few accurate lattice inputs.

Thus, QCD sum rules for the mass dimension-2 Borelized invariant amplitudes at $\tau =0$ (i.e., an infinitely large Borel mass parameter) provide an efficient tool for the analysis of the dependence of decay constants (and potentially of other hadron observables) on quark masses.

Finally, we want to mention that, besides the strong IB effect due to the up and down quark mass difference, there are other isospin violating effects due to electromagnetism, i.e. to the difference between the up and down quark electric charges. However, the inclusion of such electromagnetic corrections within a sum-rule approach is not a trivial task and it requires the development of new strategies going beyond the traditional QCD sum-rule approaches. In this respect it is worth mentioning a new lattice strategy [52] developed to deal with QCD + QED effects on quantities that require the cancellation of infrared divergences in the intermediate steps of the calculation, like, e.g., the decay rate of charged pseudoscalar mesons [53].

## Appendix: Isospin breaking in the OPE

The two-point Green function $\varPi$ of interest is given by the functional integral

43 $$\begin{aligned}&\langle T(j_1(y)j_2(0))\rangle \nonumber \\&\quad =\frac{\int D\psi (x)D\bar{\psi }(x)DA_\mu (x)\; j_1(y) j_2(0)\mathrm{e}^{i\int d^4x L(x)}}{\int D\psi (x)D\bar{\psi }(x)DA_\mu (x)\mathrm{e}^{i\int d^4x L(x)}},\nonumber \\ \end{aligned}$$

where $j_1$ and $j_2$ are (gauge-invariant) operators constructed from quark and gluon fields, and

44 $$\begin{aligned} L(x)= & {} L^{(0)}(x)-\frac{1}{2}\delta m\,\bar{q}(x)q(x),\nonumber \\ \bar{q}(x)q(x)\equiv & {} \bar{d}(x)d(x)-\bar{u}(x)u(x), \nonumber \\ \delta m\equiv & {} m_d-m_u. \end{aligned}$$

Here, $L^{(0)}(x)$ is the *SU*(2)-symmetric Lagrangian describing two equal-mass quarks, with the quark-mass term

45 $$\begin{aligned} m(\bar{d}d +\bar{u}u),\quad m\equiv \frac{1}{2}(m_d+m_u). \end{aligned}$$

Important for our argument is that the operators $j_1$ and $j_2$ do not contain light-quark masses explicitly, although they, of course, contain the light-quark field operators. For instance, one may consider

46 $$\begin{aligned} j_1(x)= & {} \bar{q}(x)\gamma _\mu \gamma _5 Q(x), \nonumber \\ j_2(x)= & {} (j_1(x))^\dagger =\bar{Q}(x)\gamma _\nu \gamma _5 q(x), \quad q=u,d. \end{aligned}$$

After expanding Eq. (43) in powers of $\delta m$, one finds

47 $$\begin{aligned} i\langle T(j_1(y)j_2(0))\rangle= & {} i\langle T(j_1(y)j_2(0))\rangle ^{(0)}-\frac{\delta m}{2}i^2\nonumber \\&\times \left\langle T\left( j_1(y)j_2(0)\int \bar{q}(z)q(z) \mathrm{d}z\right) \right\rangle ^{(0)} \nonumber \\&+O (\delta m^2), \end{aligned}$$

with the superscript “0” indicating that the full Green functions correspond to the *SU*(2)-symmetric QCD with two light quarks with degenerate mass *m*. Let us emphasize the appealing feature of the expansion (47): at each order in $\delta m$, one encounters the full Green function of the *SU*(2)-symmetric QCD.

One may expect that the power corrections in the OPE for the three-point Green function $\varGamma$ of the *SU*(2)-symmetric QCD are the *SU*(2)-symmetric condensates, e.g., $\langle \bar{u} u\rangle =\langle \bar{d} d\rangle \equiv \langle \bar{q}q\rangle$. However, in order to obtain the contributions of interest, we need to perform the limit $q\rightarrow 0$. This step cannot be done easily: the OPE for $\varGamma$ is only given by local *SU*(2)-symmetric condensates if one keeps $q^2$ large and negative; a straightforward extension of the known power corrections to $q\rightarrow 0$ leads to a wrong result: it is well known that if one naively extends power corrections in the vector three-point function to $q=0$, then they do not satisfy the Ward identity (see, e.g., [26–29]).

On the other hand, one can proceed by expanding $\varPi (p)$ in powers of the small quark mass; then the mass derivatives emerge. Translating the expression (47) into momentum space, to $O(\delta m)$ accuracy we obtain

48 $$\begin{aligned} \varPi (p)=\varPi ^{(0)}(p)-\frac{1}{2}\delta m\,\varGamma ^{(0)}(p,q=0), \end{aligned}$$

where $\varPi ^{(0)}(p)$ is the full two-point function of *SU*(2)-symmetric QCD, and $\varGamma ^{(0)}(p,q=0)$ is the three-point function of the scalar current $\bar{q}q$ at zero momentum transfer, also calculated in the full *SU*(2)-symmetric theory. Consequently, finding the leading-order *SU*(2)-breaking effects reduces to calculating the Green functions in *SU*(2)-symmetric QCD.

Now, consider the correlation functions

49 $$\begin{aligned} \varPi (p)= & {} i\int \mathrm{d}^4y \mathrm{e}^{i p y}\langle T(j_1(y)j_2(0))\rangle , \nonumber \\ \varGamma (p,q)= & {} i^2\int \mathrm{d}^4y \mathrm{d}^4z \mathrm{e}^{i p y}\mathrm{e}^{-i q z} \nonumber \\&\times \langle T(j_1(y)j_2(0)\bar{q}(z)q(z))\rangle . \end{aligned}$$

For the two-point function, we can write the dispersion representation

50 $$\begin{aligned} \varPi ^{(0)}(p)= & {} \int \limits _{(m_Q+m)^2}^\infty \frac{\mathrm{d}s}{s-p^2}\rho (s,m_Q,m) \nonumber \\&\times \theta (s-(m_Q+m))^2. \end{aligned}$$

Using the well-known relation

51 $$\begin{aligned} \varGamma ^{(0)}(p,q=0)=-\frac{\partial }{\partial m}\varPi ^{(0)}(p), \end{aligned}$$

the three-point function at zero momentum transfer may be related to the mass derivative of the two-point function, which then leads to the appearance of the mass derivatives of the quark condensate.

## Footnotes

1 The LD limit in Borel sum rules was introduced and discussed in [22–25] in connection with the pion and nucleon elastic form factors, and later applied to the analysis of meson transition form factors in [26–29]. A specific feature of this limit is the vanishing of power corrections in the two- and three-point Borelized correlation functions of axial-vector and vector currents of light quarks.

2 The second-order pseudoscalar and vector spectral densities near the threshold, Eqs. (30) and (31) in [35, 36], contain three unknown constants, $\tilde{c}_{FF}$, $\tilde{c}_{FA}$, and $\tilde{c}_{FL}$, which cancel in the ratio ${f_{V}}/{f_P}$.
